# Physical Fitness and Activity Levels among Chinese People with Schizophrenia: A Cross-Sectional Study with Matched Case-Control Comparison

**DOI:** 10.3390/ijerph17103564

**Published:** 2020-05-19

**Authors:** Daniel Bressington, Yan Li, Sabina Hulbert, Yim Wah Mak

**Affiliations:** 1 School of Nursing, The Hong Kong Polytechnic University, Hong Kong, China; Dan.bressington@polyu.edu.hk; 2 Florence Nightingale Faculty of Nursing, Midwifery and Palliative Care, King’s College London, London WC2R 2LS, UK; yan.li@kcl.ac.uk; 3 National Institute for Health Research, Research Design Service South East, Centre for Health Services Studies, University of Kent, Canterbury CT2 7NZ, UK; S.Hulbert@kent.ac.uk

**Keywords:** physical fitness, activity levels, schizophrenia, recovery, matched-control, cross-sectional

## Abstract

People with schizophrenia have an increased risk of developing cardiometabolic diseases and a reduced life expectancy. Studies conducted mainly in Western settings report low amounts of activity and poor levels of fitness in this population. This study aims to compare physical fitness and activity levels between people with schizophrenia/healthy matched controls and investigate potential associations between these variables. A cross-sectional study was conducted with 57 community-dwelling people with schizophrenia and 57 age-, gender- and body mass index (BMI)-matched controls. Participants completed the international physical activity questionnaire and the Young Men’s Christian Association (YMCA) fitness assessment protocol with accompanying cardiovascular/lung function tests. Cardiorespiratory fitness was significantly better in healthy matched controls than individuals with schizophrenia in all areas (all *p* < 0.05, *d* = 0.38 to 1.06). Performance in best trunk flexion, half sit-ups and one-minute pulse recovery following the three-min step test were significantly worse in the schizophrenia group (all *p* < 0001, *d* = 0.76 to 1.04). Higher levels of weekly moderate activity (t = −2.66, *p* = 0.009) and total weekly activity levels (t = −2.013, *p* = 0.047) were reported by the healthy controls. Levels of vigorous activity were significantly correlated with some areas of lung functioning in the schizophrenia group (all *p* < 0.05). The findings show that Chinese people with schizophrenia have significantly poorer fitness than matched healthy controls, demonstrating the need to provide timely effective exercise-based interventions as a matter of routine to attenuate the risk of developing chronic physical illnesses.

## 1. Introduction

People with severe mental illness (SMI) defined as schizophrenia, bipolar affective disorder and major depressive disorder, have about 10–20 years shorter life expectancy than the general population [1]. Cardiovascular disease (CVD) is the major cause of mortality [2]. Previous studies revealed that unhealthy lifestyles such as smoking, physical inactivity, obesity and side effects of antipsychotic medications are important risk factors of CVD [3,4]. Although CVD in people with mental illness are common, they are often diagnosed late and treatments may be inadequate [2]. Chronic physical illnesses also negatively influence quality of life among people with SMI [3,4].

Early studies have shown that among the modifiable risk factors of CVD, a lower level of physical fitness is associated with an eightfold increased risk of all-cause and CVD mortality for unfit groups when compared to their fit counterparts [5,6] and approximately double the CVD risk is found when inactive groups are compared with active groups [7]. More recent studies have also indicated that physical activity levels are associated with mortality levels independent of other risk factors in adult men [8], highlighting the importance of increasing activity levels to improve health outcomes. Indeed, there is overwhelming evidence from intervention studies conducted in the general population that increasing levels of physical activity reduce the risk of premature mortality by at least 20–30% [9]. Unfortunately, physical activity levels in people with SMI have been consistently shown to be far lower than in the general population, for example, a global systematic review and meta-analysis including over 30,000 people with SMI from 69 studies concluded that SMI participants were significantly less likely than health controls to reach recommended daily activity levels with an odds ratio (OR) of 1.5 [10]. This is particularly concerning because of the increased likelihood of people with SMI developing chronic illnesses such as CVD that are linked with low levels of activity and poor physical fitness [11].

Physical fitness can be defined as a set of independent attributes which indicate ability to perform physical activities. Cardio-respiratory fitness (CRF) is one of the components of physical fitness, it is a strong predictor of mortality levels [8] and a condition which can be measured more objectively and with greater reliability than physical activity [12]. One of the reasons for this is that physical activity is self-reported in many studies and is therefore subject to misreporting [13], leading to underestimation of the association between physical activity and health outcomes. Other components of physical fitness that indicate health status include muscular endurance, muscular strength and flexibility. These measures are particularly important for people with SMI due to the need to identify and objectively measure health inequalities in this vulnerable patient group [14].

Recommendations for monitoring the health status of people with severe mental illness are available in various countries [15]. However, the screening and assessment of physical fitness in these individuals remains unclear, even in well-developed countries [14]. Identification and assessment of the physical fitness of this group of individuals are very important for early management, reduction of mortality as well as monitoring and facilitating recovery [15,16]. For example, a systematic review and meta-analysis of 39 trials of physical activity interventions for people with mental illness concluded that such interventions resulted in significant reductions of the symptoms of depression and schizophrenia, and improvements in aerobic capacity, anthropometric measures and quality of life [17].

Assessment of physical fitness in the western general populations is well documented [12,18,19,20,21,22,23]. Yet, data relating to the physical fitness of individuals with severe mental illness are still relatively scarce [24,25,26,27], particularly in Asian populations. To the best of our knowledge, cardiorespiratory fitness, fitness performance and physical activity levels have not yet been explored and compared between Chinese people with schizophrenia and matched healthy controls. This is an important gap in understanding because evidence shows that variations in disease by different ethnic groups is related to geographical location, lifestyle, cultural and socio-economic factors [28]. Similarly, although ample studies demonstrate that people with schizophrenia have an increased risk of developing cardiometabolic diseases [29], there is a lack of evidence on the differences in fitness between Chinese healthy controls and people with schizophrenia who have not developed cardiometabolic disorders. Furthermore, it is not yet clear if and to what extent activity and physical fitness levels in people with schizophrenia who are otherwise healthy are associated with degree of recovery from schizophrenia.

### Study Objectives

1. To quantity and compare physical fitness performance, cardiorespiratory fitness and rates of physical activity in otherwise healthy individuals with schizophrenia compared with age-, gender- and body mass index (BMI)-matched healthy controls.
2. To investigate potential associations between physical fitness performance, cardiorespiratory fitness and physical activity levels in people with schizophrenia and matched healthy controls.
3. To investigate potential associations between physical fitness, physical activity levels and degree of recovery in individuals with schizophrenia.

These study objectives were designed to achieve the overall aim of identifying physical fitness disparities faced by otherwise healthy Chinese people with schizophrenia. We anticipated that the results would also help identify modifiable factors that could be incorporated into interventions designed to improve physical fitness in this patient group.

## 2. Materials and Methods

### 2.1. Study Design and Participants

A cross-sectional study was conducted with fifty-seven community-dwelling people who were diagnosed with schizophrenia and referred to out-patient settings for rehabilitation. Fifty-seven age-, gender- and BMI-matched controls were also recruited for measuring physical fitness and activity levels.

#### 2.1.1. Ethical Considerations

The potential participants with schizophrenia were screened by the mental health service locations for eligibility and safety to participate by the clinical staff in each setting. Eligible clients were given a verbal and written explanation of the purposes of the study. They were given the opportunity to ask questions and allowed at least three days to consider their participation. The information clearly stated that participation was voluntary and that all participants had the right to withdraw from the program at any time without any resulting negative influence on the services they received from the mental health service centers. Written informed consent combined in the questionnaire of self-perception of physical functional health via the Chinese translated physical activity readiness questionnaire (PAR-Q) [30] was obtained from all participants prior to conducting the physical fitness assessments. All participants were informed that they would be excluded from the main study if they failed the PAR-Q criteria. Ethical approvals for conducting this study were obtained in advance from the Human Subject Research Committee of the University (reference: HSEARS2017061003) and from each of the mental health service locations.

#### 2.1.2. Participant Recruitment and Selection

Participants with schizophrenia were recruited using a convenience sampling strategy from 10 community hostels run by the Social Welfare Department of the Government of Hong Kong or by non-governmental organizations. All locations were residential settings that provide long-term community-based accommodation and non-clinical support for adult clients with a mental disorder who are at the stage of rehabilitation. Residents need to be currently mentally stable and comply with their prescribed medication regime under supervision in order to reside in the hostels. The specific hostels involved in this study were chosen from all regions of Hong Kong (Hong Kong Island, Kowloon Peninsula and the New Territories) to ensure the representativeness of the schizophrenia sample.

The screening of the potentially eligible schizophrenia participants’ psychiatric diagnosis, physical illnesses and age was conducted by staff in the community settings. Potential participants were invited to the Integrated Health Clinic of the University or at the community settings where the participants resided for an assessment and interview. Those individuals who could complete the questionnaire, provide informed consent, follow the instructions and were able to communicate clearly and accurately were invited to join the study. Healthy control subjects were recruited from the campus users of the University (i.e., students, staff and other campus users). Purposive recruitment of the controls was performed to match the gender, age and BMI of the participants with schizophrenia. The matching was performed by independent research staff from another project who were blinded to the objectives of the present study.

#### 2.1.3. Inclusion and Exclusion Criteria

Individuals with schizophrenia were recruited into the study if they met the following criteria: (1) aged 18 years or above, (2) diagnosed with schizophrenia (according to Diagnostic and Statistical Manual of Mental Disorders, fourth edition; DSM V), (3) were referred to participating mental health service centers by a medical doctor, (4) able to communicate in Cantonese/Chinese. Individuals were excluded if they were unable to participate in a screening interview due to their mental state; if they did not have capacity to provide informed consent; if their medication regime had been revised in the last three months; reported having diagnosed cardiovascular, neuromuscular or endocrine disorders (i.e., hypertension, heart disease, arthritis, asthma, tuberculosis, chronic obstructive pulmonary disease (COPD) diabetes) or pregnancy.

Healthy matched controls were included if they (1) were aged 18 years or above, (2) reported being free of psychiatric illness, (3) reported having had received a general physical examination in the previous year prior to recruitment, (4) able to communicate in Cantonese/Chinese. They were excluded if they reported having diagnosed cardiovascular, neuromuscular or endocrine disorders or pregnancy.

Potential participants who had one or more acute illness, based on the PAR-Q [30], such as musculoskeletal problems, heart disease, hypertension, with history of lost balance of dizziness/consciousness in the 12 months prior to the assessments or who were not recommended to do exercise by the doctors and physicians were excluded.

#### 2.1.4. Sample Size Calculation

Our sample size calculation was based on the effect size taken from the average of two studies measuring physical performance and cardiorespiratory fitness of people with SMI versus healthy controls. The first is a study comparing sit-and-reach performance between individuals with bipolar disorder vs. healthy controls, which reported that the mean difference was equivalent to a moderate effect size of Cohen’s d = 0.473 [31]. The second study was a systematic review and meta-analysis (n = 310) of the cardiorespiratory fitness of people with schizophrenia compared with healthy controls that reported an effect size of Hedges’ g = −1.01. We, therefore, calculated (using G-power), the estimated total sample size required to detect a conservative effect size of d = 0.7 is 68 (i.e., 34 subjects per group), using an alpha of 0.05, a power of 0.8 with the allocation ratio as 1:1 in two-tailed *t*-test. Taking into account a potential dropout rate of about 15% [16], we needed to recruit a minimum of 40 subjects in each group.

### 2.2. Data Collection Procedures

The clients referred from the centers were screened for eligibility by a research assistant during an interview. Participants’ level of physical fitness was assessed using three tests (three-min step test, sit-and-reach test and half sit-up test). All questionnaire data and health assessment data were collected by six trained research assistants. Training for the research assistants was provided by the chief investigator. Each assistant passed a competency test for assessing physical fitness before conducting the tests for the participants.

#### 2.2.1. Demographics and Health Lifestyle Factors

All participants were interviewed guided by a structured questionnaire. In addition to the validated assessment tools, the following demographic and lifestyle information were included in the interview: (a) socio-demographic information (i.e., gender, age, education attainment, illness duration, accommodation); (b) current smoking status (c) willingness to join a regular health check-up program.

#### 2.2.2. Levels of Physical Activity

Physical activity level over the previous seven days was assessed using the Chinese version of International physical activity questionnaire (IPAQ-C; Macfarlane et al., 2006 [32]). The IPAQ-C is a self-reported measure. It has demonstrated adequate reliability and validity for the measurement of total physical activity in Chinese populations, however its results should be treated with some caution because its concurrent validity with more objective measures, such as physical activity logs and accelerometer-derived data has not been proven [32]. In accordance with the IPAQ protocol [33] we calculated a sum of total weekly metabolic equivalent (MET) physical activity in minutes per week for each intensity of activity.

#### 2.2.3. Health Checks on Body Mass Index, Blood Pressure, and Heart Rate

Before the physical fitness tests, physical indices of cardio-vascular functions such as body mass index, blood pressure, heart rate were checked. The body mass index was calculated by dividing the participants’ weight (kg) by the square of their height (m) [34]. Participants’ height was measured without the wearing of shoes, and their weight was measured when wearing light clothing. Weight and height were measured using the clinical settings standard equipment that is regularly checked and calibrated. Blood pressure (mmHg) and pulse (beats/min) was measured using automated blood pressure monitor, with two readings taken approximately 30 min apart.

#### 2.2.4. Physical Fitness Tests

Cardio-respiratory fitness (CRF) was assessed using the YMCA fitness assessment protocol and a range of accompanying cardiovascular (i.e., levels of oxygen saturation, exhaled carbon monoxide (CO) and lung function tests, forced vital capacity (FVC), i.e., the total volume of air that can be exhaled during a maximal forced expiration effort, forced expiratory volume in one second (FEV_1_); the volume of air exhaled in the first second under force after a maximal inhalation, peak expiratory flow and recovery heart rate). We also calculated the percentage of predicted value for the FEV1/FVC ratio. Lung function test was performed using the Cosmed^®^ Spiropalm spirometer (Cosmed, Rome, Italy). The technique was carried out according to the guidelines of the American Thoracic Society [35] and the reference values proposed for Asian populations. The last author or her trained delegates performed the tests. Previous studies have also adopted the YMCA Protocol using three measures [19] (1) the three-min step test, (2) the modified sit-and-reach test, and (3) the half sit-up test to measure performance of cardio-respiratory endurance, flexibility, and muscular strength and endurance, respectively. The inclusion of these measures was justifiable because the YMCA protocol is commonly used in clinical settings, and as age- and gender-based normative data are available we would be able to make direct comparisons of the participants’ physical fitness with population-based norms. Furthermore, these fitness tests have been used for people with severe mental illness [22]. Thus, the following three tests were conducted in the present study:

1. The three-min step test: participants were required to step up and down on a 30.5 cm high bench at the rate of 96 beats (24 steps) per minute for a total of three min. The test is scored as the recovery heart rate in beats per minute over 1 min, taken while seated beginning 5 s after the participant’s final step, with a higher reduction in heart rate over 1 min representing better cardiovascular fitness. Heart rate was monitored and recorded using a digital pulse oximeter.
2. Sit-and-reach by a sit and reach box to perform a trunk flexibility test (using 15 cm at the level of the feet). The score for this test is the centimetres reached, recorded as the best of three trials.
3. The half sit-up test: participants were required to raise their body to an angle of 30 degrees. The total score for this test is the total number of properly executed repetitions at the end of 1 min.

#### 2.2.5. Subjective Recovery Status (Schizophrenia Participants Only)

Recovery from mental illness was assessed using the 24 item self-rated Chinese language version of the recovery assessment scale originally devised by [36], higher total scores indicate a better level of recovery. Patients respond to each item using a five-point Likert scale (1, strongly disagree; 5, strongly agree). The Chinese version (RAS-C) [16] is reported to have good internal consistency when assessing the subjective perception of recovery among Chinese people in Hong Kong. The measure also has good concurrent validity with other validated recovery measures and good construct validity with life satisfaction, stigma, symptoms and functioning [16].

### 2.3. Data Analysis

Data were analyzed using IBM SPSS for Windows, version 23 (IBM Corp, Armonk, NY, USA). All data was screened for outliers (threshold classified as a value that falls outside of three standard deviations) and data entry errors using frequency tables for categorical variables and inspection of central tendency indexes and distributions for scale variables. Normality of distribution for scale variables was assessed referring to statistical parameters (i.e., skewness and kurtosis as well as Shapiro-Wilk and Kolmogorov-Smirnov tests) and visual inspection of histograms and Normal P-P plots. Without serious violations of the assumptions for normal distribution and a relatively large sample (n > 50), parametric tests were performed for the data analysis. Descriptive statistics (e.g., mean, standard deviation; SD) or frequencies (percentages) were used to summarize the socio-demographic/clinical information and the study variables. *T*-test/chi-square tests were performed for the comparisons of socio-demographic/clinical characteristics, physical fitness, activity levels and recovery between schizophrenia/healthy controls. Pearson’s correlation was performed to explore the correlations between study variables, and where applicable, multiple linear regression was performed with its assumptions tested. Level of statistical significance was set at *p* < 0.05.

## 3. Results

Data were collected between May 2017 to Jan 2019. Out of the 10 community-based mental health locations, we recruited 2–11 people with schizophrenia from each of the sites, an average of 6 participants were recruited from each site. While all the healthy control subjects were recruited from the University. One hundred and nineteen people with schizophrenia and 172 healthy controls were approached to take part in the study. Of these 83 people with schizophrenia (70%) and 140 (81%) of healthy controls agreed to take part. Reasons for declining participation were mainly because participants were either not available or not interested to join. Of the 83 people with schizophrenia, 26 of them were excluded based on the criteria set out by the PAR-Q (resulting in recruiting a total of 57 people with schizophrenia joining the study). Subsequently, if more than one matched healthy control was available for each schizophrenia participant, we used a random sampling method to select the healthy matched control participant used for analyses (using a random number generator).

### 3.1. Socio-Demographic and Clinical Characteristics of the Study Participants

Socio-demographic and clinical characteristics of the study participants (both schizophrenia and healthy control groups) are presented in Table 1. The total sample comprised 114 participants matched for age, gender, and BMI. The average age was 45 years (age range 19–68), with 39 Male and 18 Female participants in each of the two conditions. For 70.2% of the patients with schizophrenia the highest level of education achieved was secondary and for 15.8% of them it was tertiary or above, rather different from the 40.4% of healthy controls who had secondary as their highest level of education and 59.6% who had tertiary or above levels. While the majority of the healthy controls reported having a job or doing any unpaid work outside their home (89.5%), just below half of the patients with schizophrenia did so (49.1%). Similarly, while the majority of the healthy controls was married (64.9%) only a small minority of the patients with schizophrenia were married (14%). Participants’ accommodation status was also distinctly different between the two groups, around three-quarters (75.6%) of the healthy controls lived in their own homes, whereas a similar proportion of the patients with schizophrenia (77.2%) lived in a hostel/halfway home. Patients with schizophrenia were found to be significantly more likely to smoke than healthy controls and those patients with schizophrenia who did smoke, reported smoking a significantly higher number of cigarettes consumed a day than their healthy control counterparts. Confirming the appropriateness of the matching procedure, there were no significant differences in weight (t = −0.026, *p* = 0.979) height (t = −0.769, *p* = 0.444), BMI (t = 0.52, *p* = 0.604) and age (t = 0.487, *p* = 0.627) between the two groups.

### 3.2. Physical Fitness, Activity Levels and Recovery of Study Participants

In order to address study objective 1, we conducted a series of independent samples *t*-tests to identify significant differences in cardiorespiratory fitness, fitness performance and activity levels between the two groups.

#### 3.2.1. Physical Fitness

Cardiorespiratory fitness was found to be significantly better in healthy matched controls than individuals with schizophrenia in all areas, with effect sizes ranging from *d* = 0.38 to 1.06 (details presented in Table 2). The levels of exhaled carbon monoxide (CO) were found to be significantly higher in patients with schizophrenia (t = 2.71, *p* = 0.008). Whereas, the mean measured value for FVC was significantly higher in healthy controls (t = −2.79, *p* = 0.006), as was FEV_1_ (t = −3.18, *p* = 0.002), oxygen saturation levels (t = −1.99, *p* = 0.048) and peak expiratory flow (t = −5.47, *p* < 0.001).

Performance on the fitness testing also revealed that healthy controls were significantly fitter than people with schizophrenia with effect sizes ranging from *d* = 0.76 to 1.04. Specifically, recovery heart rate (bpm after 3-min step test) (t = 4.44, *p* < 0.001), best trunk flexion performance (t = −3.98, *p* < 0.001) and number of half sit-ups (t = −4.32, *p* < 0.001) were all significantly better in the healthy control group than the schizophrenia group.

#### 3.2.2. Activity Levels

Various measures of physical activity were obtained using the IPAQ tool. We computed total MET minutes a week for vigorous, moderate and walking equivalent activities, and total time in minutes per day spent sitting on weekdays and weekends. The independent *t*-tests revealed that participants in the two conditions report quite similar levels of physical activity, the only statistically significant differences being higher levels of weekly moderate activity (t = −2.66, *p* = 0.009, effect size *d* = 0.51) and total weekly activity levels (t = −2.013, *p* = 0.047, effect size *d* = 0.39) in healthy controls than people with schizophrenia. However, participants with schizophrenia spent significantly less time sitting on weekdays than the healthy controls (t = −2.643, *p* = 0.009, effect size *d* = 0.50).

#### 3.2.3. Recovery (Schizophrenia Group Only)

The sample mean for the total composite score is 87.44 (SDSD = 0 14.73). No significant differences were detected between male and female participants on any of the individual RAS items or total composite score.

### 3.3. Relationships between Physical Fitness, Physical Activity Levels and Degree of Recovery among Study Participants

In order to achieve Study Objective 2, correlational analyses were performed between the cardiorespiratory fitness variables and activity levels for the two separate study groups. For participants with schizophrenia, significant moderate correlations were found between total overall activity levels with FEV_1_ % predicted (r = 0.311, *p* = 0.026) and exhaled CO (r = −0.303, *p* = 0.027). Vigorous activity level was significantly associated with FVC % predicted (r = 0.319, *p* = 0.023), FEV_1_ % predicted (r = 0.382, *p* = 0.006), peak expiratory flow (r = 0.312, *p* = 0.026) and measured FEV_1_ values (r = 0.318, *p* = 0.023). Whereas weekly moderate activity level was significantly associated with exhaled CO (r = 0.312, *p* = 0.026), and FEV_1_ % predicted (r = 0.308, *p* = 0.028). For the healthy control group, a significant weak correlation was found between vigorous activity and peak expiratory flow only (r = 0.297, *p* = 0.029).

In relation to associations between fitness performance and activity levels, the schizophrenia group were only found to have a significant moderate size correlation between moderate activity level and best trunk flexion (r = 0.366, *p* = 0.008). Whereas no significant correlations were found between fitness performance and activity levels in the healthy control group. In order to address Study Objective 3, we also explored the relationships of the total recovery score with physical fitness and activity levels for the schizophrenia group, however no significant correlations were revealed.

Followed by the correlational analysis, we performed a series of regression analyses to determine the inter-relationships between participants’ activity levels and physical fitness in the whole sample (i.e., the pooled data) and the two separate groups. When pooling the data for the regression models we inserted group as a predictor variable in order to ascertain if the group accounted for a significant amount of variance in the dependent variables. Participants’ cardiorespiratory and fitness performance variables were utilized as dependent variables, and independent variables included activity levels significantly correlated with the dependent variable. After testing all the assumptions of multiple linear regression and controlling confounding effects from socio-demographic/clinical characteristics (such as smoking and educational level) none of the regression models were found significant to explain the variance in physical fitness outcomes. It is, therefore, impossible to establish a clear pattern of relationships between physiological, fitness, activity measures and recovery level in the whole sample or in participants with schizophrenia only.

## 4. Discussion

To the best of our knowledge, this study is the first to directly compare levels of physical fitness and physical activity between otherwise healthy Chinese people with schizophrenia and healthy BMI, age and gender matched controls. The study results highlight the major health disparities faced by patients with schizophrenia as they were found to have lower levels of cardiorespiratory fitness than healthy matched controls in all areas. Generally, these findings illustrate that poor cardiorespiratory fitness is likely to be common in Chinese people with schizophrenia in Hong Kong and seems to broadly concur with some earlier comparison studies conducted in other countries. For example, Ozbulut et al. (2013) [37] concluded that 60 Turkish patients with schizophrenia had low levels of cardiorespiratory fitness compared to matched healthy controls, however, the Turkish patients with schizophrenia were reported to have far higher mean levels of FVC (4.4 L) when compared to the schizophrenia group in the current study (2.95 L). Similarly, Filik et al., (2006) [38] reported that a UK sample of 602 people with schizophrenia-spectrum disorders had poor lung function when compared with the general UK population.

The lung functioning of the patients with schizophrenia in the current study is also observed to be far lower than reference values taken from over 7000 people in the general Chinese population [39], which report a mean FEV_1_ value of people aged 40 years as 3.71 (L) as opposed to a mean of 2.39 (L) for the schizophrenia group (mean age 45 years) in the current study. The current study also demonstrates that FVC in people with schizophrenia in the current study (2.95 L) is particularly poor when compared with reference values of 4.48 L for the general Chinese population of a similar average age [39]. This is despite the absence of diagnosed cardiovascular, neuromuscular and endocrine disorders in the participants with schizophrenia in the current study. Overall, our findings seem to suggest that the Hong Kong Chinese people with schizophrenia in the current study not only have poorer cardiorespiratory fitness than the matched controls, they may also have lower lung function than people with schizophrenia in other international settings. Interestingly, the FEV_1_ % predicted values were found to be moderately correlated with all levels of activity in the schizophrenia group, whereas other areas of lung function (i.e., exhaled CO or FVC % predicted) were only found to have weaker associations with some, but not all, levels of activity intensity. This finding may suggest that measuring FEV_1_ % predicted values could be an efficient and highly relevant way to profile cardiorespiratory performance and activity levels in people with schizophrenia within clinical settings. These results may also indicate that FEV_1_ % predicted values could be used as a primary respiratory-focused outcome in future studies that aim to evaluate the efficacy of interventions designed to improve activity levels in people with severe mental illness.

The fitness performance of the schizophrenia group was significantly worse that the healthy controls in relation to cardio-respiratory endurance, flexibility, and muscular strength and endurance. This finding also seems to be in line with some earlier studies conducted in other international settings; for example, the mean best trunk flexion performance for people with schizophrenia in the current study was 13.11 cm compared with 17.26 cm in healthy controls, whereas Vancampfort et al. (2016) [40] reported 17.1 cm in schizophrenia patients versus 23.5 cm in healthy controls in the UK.

We found significant differences in weekly moderate activity and total weekly activity levels between the people with schizophrenia and the healthy controls, which seems to support the findings from many previous studies. A large systematic review and meta-analysis [10] reported that people with SMI are significantly more sedentary than healthy controls (around 8 h per day during waking hours) and the pooled mean value of daily moderate or vigorous exercise in people specifically diagnosed with schizophrenia was significantly lower than healthy controls at 37 min. However, participants with schizophrenia in the current study reported far lower levels of moderate or vigorous exercise than those reported in the systematic review, with only around 77 min per week [41]. These differences may relate to variations in the geographic regions of the studies included in Vancampfort’s (2017) [10] review, for example in the studies conducted within the Oceania region the weekly pooled mean value of moderate or vigorous exercise in people with SMI was 91 min, which is broadly similar to the current study. It is possible that the activity levels of Chinese people with schizophrenia are indeed lower than other regions of the world, but larger replicate studies should be conducted in China and the wider East Asian region to establish the veracity and generalizability of our findings.

Irrespective of the potential reasons for our findings on the apparent low levels of activity, the levels of moderate or vigorous exercise in the schizophrenia group are well below the physical activity guidelines of a minimum of 150 min per week [42], placing them at risk of developing a range of chronic physical illnesses. Due to these low activity levels and evidence that increasing activity improves a range of physical and mental health outcomes [17], targeted interventions are clearly needed and should be implemented as a matter of routine. However, specially designed exercise programs and support are likely to be required for people with SMI because they face a range of barriers to engaging in exercise interventions, such as low mood, reduced motivation and a lack of social support [43].

Although no significant regression models were identified that could explain the variance in cardiorespiratory fitness or fitness performance, the bivariate correlational analyses revealed that overall vigorous activity level seemed to have a stronger relationship with various aspects of cardiorespiratory fitness than other levels of activity in the schizophrenia group. We also found that the level of moderate intensity activity was positively correlated with muscle flexibility, a result that broadly concurs with some earlier studies that have reported that activity level partially explains the variance in physical fitness performance in people with SMI [44]. It is also interesting to note that while the majority of the different aspects of physical fitness were worse in people with schizophrenia than in healthy controls in the current study, the mean difference in total overall weekly activity levels was only around 70 min and the amount of time spent sitting on weekdays was significantly higher in the control group than the schizophrenia group. Overall, these findings seem to highlight the importance of promoting vigorous intensity exercise in this patient group, particularly as earlier studies have concluded that higher improvements in cardiorespiratory functioning resulted from interventions that adopted high intensity exercise programs with high frequency [40].

The subjective recovery scores of the schizophrenia participants (as measured using the RAS) were found to be similar to some other individual studies conducted with people with SMI in other international settings, suggesting that participants in the current study were on the whole relatively hopeful and confident in relation to their recovery. A wide range of mean total RAS scores of 75.36 to 98.8 (with an overall average of 90.72) have been reported in an earlier systematic review including 28 studies [45]. Although direct comparisons are complicated by the varying sampling strategies and different population characteristics of earlier studies, the total mean score of 87.44 in the current study is somewhat higher than the mean of 82.11 reported in study of 301 community and inpatient based Japanese people with severe mental illness, but lower than the mean of 89.16 in a sample of 76 Italian inpatients with schizophrenia [36].

Based on some earlier studies that reported significant relationships between recovery level and physical health state [46] and that improvements in exercise are associated with greater psychosocial functioning [43], we hypothesized that levels of cardiorespiratory fitness, fitness performance and activity would significantly explain some of the variance in mean recovery total scores. However, this was not the case in the current study. Potential reasons for a lack of relationship between recovery score and fitness/activity could be because such a relationship actually does not exist or may be due to the generally high recovery scores reported in this study and the associated lack of score variability. Either way, further studies would be useful to establish if activity and fitness may influence subjective recovery scores in people with schizophrenia.

### Study Limitations

A number of methodological limitations should be considered when interpreting the results of this study. The convenience sampling strategy for individuals with schizophrenia and recruitment from only community-based rehabilitation treatment centers reduces the representativeness of the sample and hence the generalizability of findings to the wider Chinese schizophrenia population. The generalizability is also limited by the inclusion of stable and medication adherent participants with schizophrenia who had not been diagnosed with any cardiovascular, neuromuscular and endocrine disorders. Although all the data collectors received adequate training and were closely supervised, we did not formally test inter-rater reliability and therefore there may have been some variability in their assessments. In addition, the physical activity levels were self-reported rather than objectively recorded, and this introduces risks of recall and social desirability biases. Future replication studies should consider measuring activity more objectively in order to be more certain of the findings (i.e., using wearable actigraphy devices). When considering the statistical significance of our results, readers should appreciate that we made a decision not to adjust the significance level (i.e., using a Bonferroni correction or similar) despite conducting multiple comparisons. This was because we conducted multiple simple tests and the results of the individual tests are viewed as important, rather than considering that a single significant result rejects a universal null-hypothesis [47,48]. Therefore, instead of applying a correction to the level of significance we have provided the Cohen’s *d* effect sizes for significant results in order that the magnitude of effect is clear. Finally, given the cross-sectional nature of the study it is not possible to infer causality between the variables.

## 5. Conclusions

The study findings highlight that otherwise healthy Chinese people with schizophrenia have significantly poorer cardiorespiratory fitness, muscular strength and muscular flexibility than age gender and BMI matched healthy controls. The results may also suggest that Hong Kong Chinese people with schizophrenia have worse physical fitness than their counterparts in some other international settings. These findings provide additional confirmatory evidence that people with schizophrenia have a high risk for poor physical fitness when compared to healthy controls. The results also provide novel evidence that these fitness disparities exist in people with schizophrenia even when they have not been diagnosed with cardiovascular, neuromuscular and endocrine disorders, hence demonstrating the need to provide timely effective exercise-based interventions as a matter of routine to attenuate the risk of developing chronic physical illnesses in future.

## Figures and Tables

**Table 1:** Sociodemographic/clinical information among participants with schizophrenia vs. healthy controls.

	Schizophrenia	*N*	Healthy Controls	*N*	t/Chi-Square	*p*
Age (mean and SD)	46.33 (9.89)	56	45.32 (10.42)	57	0.487	0.627
Weight (mean and SD)	66.39 (12.15)	54	66.45 (11.89)	57	−0.026	0.979
Height (mean and SD)	164.63 (8.94)	54	165.72 (7.35)	57	−0.769	0.444
BMI (mean and SD)	24.50 (4.09)	54	24.12 (3.53)	57	0.52	0.604
Resting diastolic BP (mean and SD)	72.32 (28.07)	56	71.93 (22.20)	57		
Current smoker (-%.):						
Yes	31.6	18	12.3	7	6.20	0.022 *
No						
Number of cigarettes smoked per day (m and s.d)	16.61 (9.63)	18	7.67 (4.18)	6	2.18	0.016 *
Duration of schizophrenia diagnosis in months (mean and SD)	223.92 (146.57)	40	-	-	-	-
Willingness to join a fitness training program (%)					3.34	0.068
No	38.6	22	22.8	13		
Yes	61.4	35	77.2	44		
Level of education (%):					27.62	<0.001 **
Primary or below	14.0	8	0	0		
Form 1–Form 3	24.6	14	10.5	6		
Form 4–Form 7	45.6	26	29.8	17		
Tertiary or above	15.8	9	59.6	34		
Marital status (%):					32.95	<0.001 **
Single	73.7	42	28.1	16		
Married	14.0	8	64.9	37		
Widowed	0	0	1.8	1		
Divorced/Separated	12.3	7	5.3	3		
Accommodation (%):					79.60	<0.001 **
Hostel/halfway-home	77.2	44	24.6	14		
Public housing	17.5	10	14.0	8		
Home ownership scheme	1.8	1	54.4	31		
Private housing	3.5	2	7.0	4		
Employment status (%):					25.03	<0.001 **
Unemployed	35.1	20	1.8	1		
Retired	15.8	9	8.8	5		
Employed	49.1	28	89.5	28		

Note: BMI = body mass index, BP = blood pressure. * *p* < 0.05, ** *p* < 0.001.

**Table 2:** Physical fitness, activity levels, and degrees of recovery among participants with schizophrenia vs. healthy controls.

Variable	Schizophrenia Mean (SD)	*N*	Healthy Controls Mean (SD)	*N*	t	*p*-Value	Cohen’s *d*
**Cardiorespiratory fitness**							
Resting pulse (bpm)	79.9 (13.53)	54	71.19 (8.62)	57	3.65	<0.001 ***	0.77
SaO2	97.41 (0.98)	54	97.79 (1.03)	57	−1.99	0.048 *	0.38
Exhaled CO	5.85 (7.56)	53	2.67 (4.05)	55	2.71	0.008 **	0.52
FVC	2.97 (0.97)	54	3.44 (.84)	57	−2.79	0.006 **	0.51
FVC % of predicted value	81.72 (19.42)	54	93.21 (12.84)	57	−3.694	<0.001 ***	0.69
FEV_1_	2.41 (0.74)	54	2.82 (0.63)	57	−3.13	0.002 **	0.59
FEV_1_ % of predicted value	80.09 (19.48)	54	92.28 (13.86)	57	−3.814	<0.001 ***	0.72
Peak expiratory flow	4.97 (2.09)	51	7.15 (2.03)	56	−5.47	<0.001 ***	1.06
**Fitness performance**							
Best Trunk Flexion (cm)	13.11 (6.02)	51	17.26 (4.82)	57	−3.98	<0.001 ***	0.76
Half Sit-Ups (number)	14.77 (12.84)	47	27.21 (15.87)	56	−4.32	<0.001 ***	0.86
One-minute pulse recovery following 3-min step test (bpm)	17.94 (9.10)	34	27.38 (9.01)	42	−4.52	<0.001 ***	1.04
**Activity Levels (minutes per week)**							
Vigorous total	28.43 (60.09)	54	44.55 (59.22)	55	−1.41	0.161	
Moderate total	48.78 (68.07)	54	86.64 (79.95)	55	−2.66	0.009 **	0.51
Walking total	84.11 (98.12)	54	99.60 (82.91)	55	−0.98	0.375	
Overall activity level	161.33 (180.97)	54	230.78 (179.24)	55	−2.013	0.047 *	0.39
Time spent sitting (per weekday)	245.37 (220.76)	54	351.05 (200.44)	57	−2.643	0.009 **	0.50
Time spent sitting (per weekend day)	251.70 (233.74)	54	278.42 (168.93)	57	−0.693	0.490	
**Recovery level**							
RAS Total	87.44 (2.04)	52	-	-			

Note: FVC = forced vital capacity; FEV_1_ = forced expiratory volume in 1 s^.^ RAS = recovery assessment scale. * *p* < 0.05, ** *p* < 0.01, *** *p* < 0.001.
